# Interplay between oxidative stress, SIRT1, reproductive and metabolic functions

**DOI:** 10.1016/j.crphys.2021.03.002

**Published:** 2021-03-27

**Authors:** Faiza Alam, Hareem Syed, Sofia Amjad, Mukhtiar Baig, Taseer Ahmed Khan, Rehana Rehman

**Affiliations:** a Department of Physiology, University of Karachi, Karachi, Pakistan; b Department of Cardiology, Tabba Heart Institute, Karachi, Pakistan; c Department of Physiology, Ziauddin University, Karachi, Pakistan; d Department of Clinical Biochemistry, Faculty of Medicine, Rabigh, King Abdulaziz University, Jeddah, Saudi Arabia; e Department of Biological and Biomedical Sciences, Aga Khan University, Karachi, Pakistan; f PAPRSB Institute of Health Scienecs, Universiti Brunei Darussalam, Bandar Seri Begawan, Brunei

**Keywords:** Infertility, SIRT1, Oxidative stress, Metabolic changes

## Abstract

Silent information Regulators (SIRT1) gene stimulates antioxidants’ expression, repairs cells damaged by oxidative stress (OS), and prevents the cells’ dysfunction. In particular, the role of different Sirtuins, particularly SIRT1 in reproduction, has been widely studied over the past decade. Decreased SIRT 1 causes mitochondrial dysfunction by increasing Reactive Oxygen Species (ROS), lipid peroxidation, and DNA damage in both male and female gametes (Sperms and Oocytes), leading to infertility. In the female reproductive system, SIRT1 regulates proliferation and apoptosis in granulosa cells (GCs), and its down-regulation is associated with a reduced ovarian reserve. SIRT1 also modulates the stress response to OS in GCs by targeting a transcription factor vital for ovarian functions and maintenance.

ROS-mediated damage to spermatozoa’s motility and morphology is responsible for 30–80% of men’s infertility cases. High levels of ROS can cause damage to deoxyribo nucleic acid (DNA) in the nucleus and mitochondria, lipid peroxidation, apoptosis, inactivation of enzymes, and oxidation of proteins in spermatozoa. SIRT 1 is a cardioprotective molecule that prevents atherosclerosis by modulating various mechanisms such as endothelial injury due to impaired nitric oxide (NO) production, inflammation, OS, and regulation of autophagy. SIRT 1 is abundantly expressed in tubular cells and podocytes. It is also found to be highly expressed in aquaporin 2 positive cells in the distal nephron suggesting its involvement in sodium and water handling. SIRT1 improves insulin resistance by reducing OS and regulating mitochondrial biogenesis and function. It also decreases adiposity and lipogenesis and increases fatty acid oxidation. So, its involvement in the multiple pathways ensures its unique role in reproductive and metabolic derangement mechanisms.

## Highlights

• SIRT1 activates the defensive-signal cascade that plays as the frontline defense against ROS in human reproductive organs.
• SIRT1 deficiency cause cigarette smoking induced cell injury, insulin secretion and glucose homeostasis through PGC-1a.
• SIRT1 increase endothelial NO production by activating eNOS leading to vasodilation reversal of endothelial dysfunction.

## 1 Introduction

**Fig. 1:**
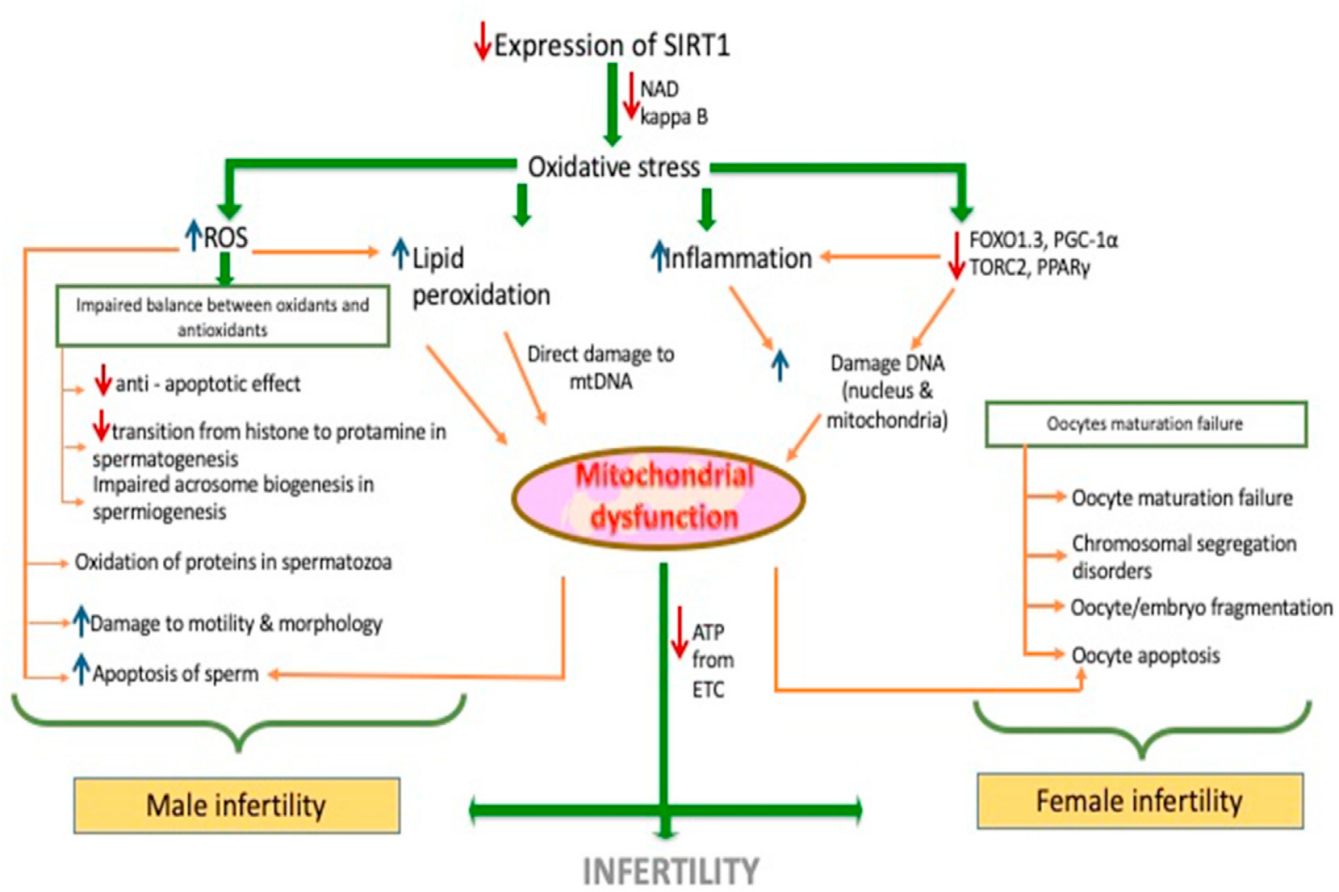
**Effect of decreased expression of SIRT1 on infertility:** Decreased expression of SIRT1, causing mitochondrial dysfunction by increase in Reactive Oxygen Species (ROS), lipid peroxidation and DNA damage in both male and female gametes (Sperms and Oocytes) leading to infertility.

Sirtuins (NAD dependant-deacylase) are involved in the deacetylation of histones and transcriptional factors regulating the cell cycle, resistance to oxidative stress, and metabolism. They are located in all essential parts of the cell, including the nucleus, cytoplasm, and mitochondria (Morris, 2013; Merksamer et al., 2013). SIRT1 has been called the “sensor” and “guardian of the redox state” in granulosa cells and oocytes(3). A vast range of cellular activities is controlled by SIRT1, including programmed cell death, autophagy, cell migration, and differentiation. This review aims to gather information about crosstalk between SIRT1 and OS, reproductive and metabolic functions (Table 1, Fig. 1).

**Table 1:** Alterations in the physiological mechanisms by decreased SIRT1 expression.

Effects of decreased SIRT1	Mechanism	Reference
SIRT1, OS and female infertility		
Decreased Ovarian Reserve	SIRT1 regulates proliferation and apoptosis in granulosa cells (GCs) and its down-regulation is associated with a reduced ovarian reserve	Tatone C et al. (Tatone and Di Emidio, 2015)
Increase in Stress	Hindrance of modulation of stress response to oxidative stress in GCs by targeting FOXL2, a transcription factor vital for ovarian functions and maintenance.	Benayoun et al. (Benayoun et al., 2011)
Increased insulin resistance	SIRT1 improves insulin resistance by reducing oxidative stress and regulating mitochondrial biogenesis and function.	Zhang HH et al. (Zhang et al., 2015)
Dysregulation of Glucose metabolism	SIRT1 regulates hepatic glucose metabolism by interacting with and deacetylating PGC-1a, which is a main transcriptional co-activator that regulates glucose metabolism in the liver at the level of gene transcription	Rodgers JT et al. (Rodgers et al., 2005)
Increased obesity	SIRT1 decreases adiposity and lipogenesis and increases fatty acid oxidation by repressing the PPARγ, inhibiting the CRTC2 or TORC2 and deacetylating and activating the PGC-1α	Picard et al. (Bordone et al., 2006); Nemoto et al., (Nemoto et al., 2005); Kilic et al. (Kilic et al., 2015)
Increased atherosclerosis (cardiovascular disease)	SIRT 1 is a cardioprotective molecule that prevents atherosclerosis by modulating endothelial injury due to impaired nitric oxide (NO) production, inflammation, oxidative stress and regulation of autophagy.	Donato et al. (Donato et al., 2015)
Renal Injury	SIRT1 inhibits sodium reabsorption in the inner medullary collecting ducts by repressing the transcription of the epithelial sodium channel, down regulates angiotensin II type 1 receptor in vascular smooth muscle cells and also promotes the resistance of RMICs to oxidative stress and injury through its anti-oxidative properties	Zhang D et al. (Zhang et al., 2009)
Accelerated oocyte ageing	Recently, SIRT1, SIRT2 and SIRT3 have emerged as protectors of oocyte against postovulatory aging. It also delays postovulatory oocyte aging through its anti-oxidative actions and by improving mitochondrial function.	Tatone C et al. (Tatone et al., 2018a)
SIRT1, OS & Male Infertility		
Decrease spermatozoa protection	SIRT1 protects spermatozoa from apoptosis. Thus, deficiency of SIRT1 leads to decrease in number of spermatozoa: 1.By ubiquitination and subsequent degradation of the transcription factor FOXO3a 2.By decreasing the caspase 3 and 9, thus reducing the caspase mediated apoptosis	Wang et al. (Wang et al., 2015); Li et al. (Li et al., 2016); Zhou et al. (Zhou et al., 2017)

## 2 Mechanism of action of sirtuins

Sirtuins have been found to affect the reproductive physiology of females by acting as antioxidants against the ROS. They act as sensors and protectors of the redox environment within the ovarian granulosa cells. SIRT1, expressed at small concentrations in oocytes, is a chief participant during oogenesis that extends to metaphase II (MII). SIRT2, chiefly located in the cytoplasm, transitorily migrates to the nucleus to control chromatin condensation and regulate the cell cycle. SIRT3 functions during fertilization and the primary embryonic growth by participating in energy metabolism (Kawamura et al., 2010). SIRT5 prevents the cell from oxidative damage by activating the enzymes which are NADPH-producing. SIRT6 is associated with telomere stability; however, SIRT7 co-activates ribosomal DNA transcription.

SIRT1 is also known to repair cells that have been damaged due to OS, thus preventing dysfunction of ovarian cells (Pillarisetti, 2008); it does this by deacetylation of members of the forkhead-box transcription factors (FOXO) family (Brunet et al., 2004). SIRT1 also promotes mitochondrial biogenesis by activating the peroxisome proliferator-activated receptor co-activator PGC1-α in the presence of NAD (Nakao et al., 2019). SIRT1 utilizes visfatin for accomplishing its effect against the oxidants. Visfatin is known to be an essential element in regulating the oxidative environment of the oocytes. However, SIRT1 is also responsible for regulating the function and expression of MnSOD (Peck et al., 2010).

## 3 SIRT1, OS, and stress

A recent study stated that Proopiomelanocortin (POMC) neuron speciﬁc ablation of SIRT1 did not change POMC, Adrenocorticotropic hormone (ACTH), and alpha-melanocyte-stimulating hormone (αMSH) levels (Ramadori et al., 2010; Yamamoto and Takahashi, 2018). The pro-CRH is acted upon by prohormone convertases 1 and 2 (PC1 and PC2) post-translationally. In the Para Ventricular Nuclei (PVN), SIRT1 elevates the PC2 levels, which in turn increases the concentration of active CRH, stimulating the HPA axis (Toorie et al., 2016). There is a probability that SIRT1 indirectly controls the HPA axis by regulating the PC1 and PC2 concentrations. Moreover, Resveratrol also augmented the expression and prolongation of the half-life of P450scc in the suprarenal gland, resulting in the increase of cortisol secretion from the adrenal cortical sites (Yamamoto and Takahashi, 2018; Li et al., 2012).

Infertile couples are stressed because of the situation, and thus, their stress hormones are higher than normal. These disturbed hormones have an indirect effect on the HOP axis via OS, causing infertility. However, the cascade of high-stress hormones could be triggered by conditions other than infertility and can lead to subfertility. SIRT1 deficiency could be one reason.

## 4 SIRT1, OS and female reproduction

In particular, the role of different Sirtuins, particularly SIRT1 in reproduction, has been widely studied over the past decade. In the female reproductive system, SIRT1 regulates proliferation and apoptosis in granulosa cells (GCs), especially during the follicular atretic phase, and its down-regulation is associated with a reduced ovarian reserve (Tatone et al., 2018a). Porcine GCs transfected with SIRT1 showed increased proliferation markers proposing the role of SIRT1 in GC differentiation and luteinization (Sirotkin et al., 2014). SIRT1 modulates the stress response to OS in GCs by targeting FOXL2, a transcription factor vital for ovarian functions and maintenance (Benayoun et al., 2011).

It also delays postovulatory oocyte aging through its anti-oxidative actions and by improving mitochondrial function (Liang et al., 2018). SIRT1 is highly expressed in the hypothalamus, particularly in the GnRH neurons (Di Sante et al., 2015). Its role in the HPG axis has been identified in knockout mice, which had decreased GnRH expression and, in turn, decreased levels of both FSH and LH (Kolthur-Seetharam et al., 2009a). Hypogonadotropic hypogonadism has also been reported in such mice due to GnRH neuronal migration (Di Sante et al., 2015).

Increasing SIRT1 activity can improve fertility by conserving ovarian reserve, regulating proliferation and apoptosis of GCs, and protecting against OS. Sirtuin-mediated regulation of energy homeostasis, mitochondrial biogenesis, and chromatin remodeling can further be explored to identify its effectiveness in females with PCOS, diabetes, endometriosis, xenobiotic stress, and aging. Sirtuin-based signaling can, therefore, be applied as diagnostic tools and potential targets for therapeutic applications in reproductive medicine (Tatone et al., 2018a). Regulation of insulin sensitivity and maintenance of the energy balance (Xiong et al., 2011) via various pathways renders SIRT1 different from other family members.

## 5 SIRT 1, OS and oocytes/ovaries

SIRT1 plays its role as an effective modulator in mammals’ oocytes at diverse phases of follicle maturation by modifying mitochondrial mechanisms to synthesize energy (13, 20). Within the oocytes, the mitochondrial enzymes (including MnSOD) are deacylated by SIRT1(21, 22).

This has already been proven that SIRT1 demonstrates a front-line defense mechanism against the ROS through the FoxO3a-MnSod axis in the mouse’s immature ovarian oocytes at the germinal vesicle phase (GV) (Di Emidio et al., 2014).

These conclusions validate SIRT1 as a principal and precise pharmacological focus against ROS destroying oocytes (Liu et al., 2012). They also propose SIRT1 to be a pharmacological excitation that might be an appropriate line to advance to treat improved oocyte achievement during OS-mediated physiological disruptions, especially in early aged ovaries (Di Emidio et al., 2014).

## 6 SIRT1, OS, and subfertility

Nicotinamide and acetyl group of substrate reacts enzymatically in the presence of SIRT1, forming O-acetyl ADP ribose as a metabolite. This reaction generates NAD with helps the ovarian cells to grow (Pillarisetti, 2008).

In mice model, Sirtuin regulation has been related to fertility (McBurney et al., 2003). Mice strains with Sirtuin deficiency demonstrate a tiny phenotype with growth-related defects and early postnatal expiries (Coussens et al., 2008). In cases of SIRT1 deficiency, Resveratrol, an indirect SIRT1 activator, demonstrated a vital role of SIRT1 as an activator of steroidogenesis during luteinization and differentiation of granulosa cells (Morita et al., 2012).

## 7 SIRT 1, OS and mitochondria

Cytochrome P450, NADPH oxidase family (28) and mitochondria generate ROS during the physiological electron transport chain reactions. Mitochondrial ability to maintain the ROS is achieved by the total redox environment and its compartmentalized reduction activity, which is dependent on the antioxidant buffers (electron donors) (NADPH, NADH and glutathione) (Miranda-Vizuete et al., 2000). However, the antioxidants like manganese superoxide along with peroxides and mitochondrial thioredoxin cycle system counter balances the negativity. This stability is very much demanded for the maturation of ovarian follicles; otherwise, the effects are detrimental (30). SIRT1 inhibition leads to an inability of the oocyte to up-regulate SOD2 and counteract the increase in ROS under increased OS(23).

Manganese superoxide converts the injurious free radicals (superoxides), which are capable of damaging the DNA, to oxygen molecule and hydrogen peroxide. Further, catalase converts hydrogen peroxide to water and oxygen. Normal oxidant generation is essential for various physiologic functions of the reproductive system (ovary), including ovarian steroid genesis, oocyte maturation, ovulation, blastocysts formation, implantation, luteolysis and luteal maintenance in pregnancy. However, change in this normality becomes another important regulator of ovarian germ cell growth and stromal cell physiology (31).

## 8 SIRT1, oxidative stress, and male infertility

Sperm cells are highly vulnerable to oxidative damages as the testicular tissue has an increased rate of cell division, mitochondrial oxygen and unsaturated fatty acid consumption, and low oxygen pressure due to the weakness of the testicular artery (Asadi et al., 2017). SIRT1 has also been found to have a significant role in the male reproductive system. SIRT1 is involved in spermatogenesis by influencing specific functions of male germ cells, Sertoli cells, and Leydig cells (Tatone et al., 2018b). Most of these effects are due to the antioxidant effect of SIRT1 in spermatogenesis. Mostefa et al. reported OS and SIRT1 deficiency as the cause of male infertility in patients with varicocele. As varicocele also causes oxidative stress; however, the deficiency of SIRT1 has added effect of decreased seminal antioxidant defenses (Agarwal et al., 2015; Mostafa et al., 2015). The low expression of seminal SIRT1 expression has significant positive correlations with concentration, total motility, and normal morphology of sperm (Mostafa et al., 2018). There are many mechanisms by which SIRT1 modulates the antioxidant outcomes. One of these antioxidant effects is that it protects spermatozoa from apoptosis by hydrogen peroxide via ubiquitination and subsequent degradation of the transcription factor FOXO3a (Wang et al., 2015; Li et al., 2016). SIRT1 also exerts an anti-apoptotic effect by deacetylation of lysine residues on proteins, affecting their functions, including transcriptional activity, DNA binding, protein binding, protein stability, and translocation. SIRT1 also inhibits the microglial-derived factors via the p53-caspase-3-dependent mechanism, thus abolishing the caspase-mediated apoptosis (Zhou et al., 2017; Ye et al., 2013). Resveratrol reverses this effect.

In addition to this, SIRT1 increases the expression of Bcl-2 and decreases the expression of BAX, thus regulating the mitochondrial membrane permeability, mitochondrial function, and cytochrome *c* release (Zhou et al., 2017; Leber et al., 2007). This is the mechanism through which decrease expression of SIRT1 affects sperm motility.

An increase in SIRT1 expression also increases nitric oxide synthase activity in vascular endothelial cells (ECs) and maintains microenvironment homeostasis, nutrient exchange, host defense reactions, and vasodilation. The mechanisms underlying this protective effect involve Sirt1/FOXOs, Sirt1/NF-*κ*B, Sirt1/NOX, Sirt1/SOD, and Sirt1/eNOs pathways (Nakata et al., 2012; Zhang et al., 2017). SIRT1 plays a significant role in regulating autophagy, a stress-induced catabolic process, by increasing mitochondrial metabolism (Ou et al., 2014). SIRT1 also has a physiological function in acrosome biogenesis during spermiogenesis by modulating autophagic flux (Liu et al., 2017). SIRT1selectively activates LC3, a key initiator of autophagy. LC3 controls major steps in the autophagic pathway, including autophagic membrane growth, autophagic cargo recognition, and the fusion of autophagosomes with lysosomes (Huang et al., 2015). Depletion of SIRT1 in germ cells causes accumulation of acetylated LC3 in the spermatid nucleus, which affects acrosome biogenesis. These spermatogenesis changes lead to an increased proportion of abnormal spermatozoa in the SIRT1-deficient mice (Liu et al., 2013).

Li et al. suggested that SIRT1 and signal transducer and activator of transcription 3 (STAT3) have a synergistic effect against oxidative stress (Li et al., 2015). SIRT1 is required for regulating normal postnatal testicular development and spermatogenesis through hypothalamus-pituitary gonadotropin (HPG) signaling (Kolthur-Seetharam et al., 2009b). SIRT1 is required for histone to protamine transition and altered chromatin condensation. Ultimately, this leads to decreased fecundity (Bell et al., 2014).

It appears that many mechanisms are underlying the function of SIRT1 against oxidative stress in spermatozoa. The low expression of seminal SIRT1 expression has significant positive correlations with concentration, total motility, and normal morphology of sperm.

## 9 Metabolic effects of SIRT1

### 9.1 SIRT1, OS and glucose metabolism

SIRT1 affects insulin secretion and glucose homeostasis through its interaction with PGC-1a. (Rodgers et al., 2005). PGC-1a is one of the main transcriptional activators of glucose metabolism in the liver. SIRT1 has been shown to deacetylate PGC-1a and hence regulate hepatic glucose metabolism. Experiments on mice pancreatic beta cells have shown that SIRT1 up-regulates insulin secretion in response to glucose stimulation and enhances glucose tolerance (Moynihan et al., 2005). Also, SIRT1 knockout mice display lower serum glucose levels than wild-type mice (Bordone et al., 2006). SIRT1 also reduces obesity by suppressing PPARg, which leads to reduced fat deposition in adipocytes (Picard et al., 2004). This, in turn, reduces obesity, which leads to the resolution of insulin resistance and type 2 Diabetes.

Insulin resistance and obesity are two characteristic features of PCOS, an endocrine disorder commonly found in reproductive-age females (Tao et al., 2015). Compared to fertile females, females with PCOS have a higher BMI and lower levels of SIRT1. Rehana et al. hypothesized that the use of Metformin in PCOS patients might act by the correction of OS caused by SIRT1 malfunction (Rehman et al., 2018). Adipose tissue can also secrete angiotensin II, which stimulates Nicotinamide Adenine Dinucleotide Phosphate (NADPH) oxidase activity, the major ROS production route in adipocytes (Hukshorn et al., 2004). Thus, it can be concluded that females with low levels of SIRT1 cannot counter the excess oxidant load produced by additional adipocytes and, consequently, develop increased OS.

### 9.2 SIRT 1, OS and obesity

Obesity is defined as a pathological increase in body fat. It is characterized by an imbalance between the intake and expenditure of calories (Mutch and Clément, 2006). Various molecules have been involved in the pathogenesis of obesity, of which SIRT1 has been an important one. SIRT1 decreases adiposity and lipogenesis and increases fatty acid oxidation by repressing the peroxisome proliferator-activated receptor-gamma (PPARγ), inhibiting the CREB-regulated transcription co-activator 2 (CRTC2 or TORC2) and deacetylating and activating the peroxisome proliferator-activated receptor-gamma co-activator 1-alpha (PGC-1α) (Picard et al., 2004; Nemoto et al., 2005; Kilic et al., 2015). Various studies have demonstrated low-grade, chronic inflammation in obesity, and this inflammation is the result of both macrophage recruitment and production of ROS (Xu et al., 2003). SIRT1 alters these macrophages’ recruitment and polarization into adipose tissues by modulating several adipokines’ expression and secretion and decreasing the risk of obesity (Hui et al., 2017).

Extremes of body weight negatively affect females’ fecundability and adversely impacts fetuses and embryos through oxidative mechanisms. Moderate exercise may assist obese women in reducing weight and restoring their fertility. Lifestyle factors such as maternal smoking, alcohol consumption, and use of recreational drugs stimulate the production of unfavorable amounts of ROS, leading to OS, which renders physiological processes of female reproduction and the fetus vulnerable to oxidant-induced damage. Exposure to environmental pollution can also give rise to excessive OS during pregnancy and has increasingly raised concern about the impact of pollutant exposure on maternal and fetal health.

### 9.3 SIRT1, OS and insulin resistance

Insulin resistance is defined as the resistance of target tissues such as skeletal muscles, adipocytes, and liver to insulin stimulation and is most often implicated in developing type II diabetes mellitus (Zhang et al., 2015). Various mechanisms of the pathogenesis of insulin resistance have been proposed, of which mitochondrial dysfunction has been a popular one. Mitochondrial abnormalities, particularly mitochondrial complex 1 lead to increased reactive oxygen species production (ROS) (Szendroedi et al., 2011). Increased ROS leads to increased oxidative stress, impairs both B cell function and insulin signaling, thereby accelerating insulin resistance (Gerber and Rutter, 2017; Morgan et al., 2007). SIRT1 restores mitochondrial complex 1 activity via the SIRT1-SIRT3-mitochondrial complex 1 pathway and alleviates mitochondrial dysfunction and OS, improving insulin resistance (Zhang et al., 2015). SIRT1 also reduces OS by overexpression of anti-oxidative enzymes, including MnSOD and Catalase (St-Pierre et al., 2006). Thus, SIRT1 may improve insulin resistance by reducing oxidative stress and regulating mitochondrial biogenesis and function.

### 9.4 SIRT 1, OS and cardiovascular disease

Cardiovascular disease due to atherosclerosis is the leading cause of death worldwide (Go et al., 2013). SIRT 1 is a cardioprotective molecule that prevents atherosclerosis by modulating the various mechanisms involved in its pathogenesis, namely endothelial injury due to impaired nitric oxide (NO) production, inflammation, OS, and regulation of autophagy (Donato et al., 2015). SIRT1 increases endothelial NO production by activating eNOS, leading to vasodilation and reversing endothelial dysfunction (Ota et al., 2010). SIRT 1 also suppresses inflammation by downregulating NF-κB activity through deacetylation (Kitada et al., 2016). SIRT 1 is noted to promote autophagy via AMPK activation, which reduces OS and inflammation and suppresses foam cell production, thereby hindering atherosclerosis progress (Luo et al., 2019). Lastly, SIRT1 reduces OS, a major causative factor of atherosclerosis, by its interplay with the FOXO transcription factors and up-regulation of anti-oxidative enzymes (Luo et al., 2014). Aging is an independent risk factor for CVD. Thus, SIRT1 acts as a cardioprotective molecule by protecting the heart from aging and ischemia/reperfusion injury, resists hypertrophic and oxidative stresses, inhibits cardiomyocyte apoptosis, and regulates cardiac energy metabolism.

### 9.5 SIRT1, OS and related renal physiology

Sirtuins, including SIRT1 present in the renal system, support the production of sufficient energy throughout the different tubular and glomerular compartments to carry out all these processes (73). SIRT 1 is abundantly expressed in tubular cells and podocytes. It is also found to be highly expressed in aquaporin 2 positive cells in the distal nephron of rats, suggesting its involvement in sodium and water handling (Zhang et al., 2009). SIRT1 inhibits sodium reabsorption in the inner medullary collecting ducts by repressing the epithelial sodium channel’s transcription (eNAC) (Zhang et al., 2009). SIRT1 also acts on the renin-angiotensin system; its overexpression downregulates angiotensin II type 1 receptor (AT1R) in vascular smooth muscle cells (Miyazaki et al., 2008).

In contrast, a reduced expression of SIRT1 is associated with the increased transcription of AT1R in podocytes (Chandel et al., 2017). The renal medulla is a high OS zone; SIRT 1 has been shown to promote the resistance of renal medullary interstitial (RMICs) cells to oxidative stress and injury through its anti-oxidative properties (He et al., 2010). One of the mechanisms through which it does so is by increasing COX-2 and PGE2 production in RMICs (He et al., 2010). Hence, in conditions that cause ureteral obstruction, SIRT1 overexpression protects the medulla from the increased oxidative stress-induced inflammation and fibrosis by induction of COX2. SIRT1 also plays a protective role in acute kidney injury; increased ROS and mitochondrial damage are necrosis features in AKI. SIRT1 activation can alleviate the increased OS and mitochondrial dysfunction, thereby protecting the kidney against AKI-mediated necrosis (Hasegawa et al., 2010; Guan and Hao, 2016). Thus, renal pathologies could result from SIRT1 mutations due to uncontrolled ROS produced by the renal tissue’s highly functional mitochondria. Evaluation of SIRT1 mutation becomes an important factor in patients with recurrent renal pathologies.

## 10 Conclusion

It seems that SIRT1 has multiple roles in human pathophysiology and biochemical pathways, from its involvement in reproduction to several metabolic functions. Further research is needed to confirm its exact role in the body’s various mechanisms. There is a need to design such pharmacologic interventions that affect SIRT1 activity to bring it back to an optimal level. So, many deleterious conditions or metabolic derangements due to SIRT1 can be prevented, treated, or at least reduced in future.

## Funding

None.

## CRediT authorship contribution statement

**Faiza Alam:** Conceptualization, Writing – original draft, designed, drafted the review, contributed to the artwork, drafted the paper, All authors approved the final version of the manuscript. **Hareem Syed:** Writing – original draft, drafted the paper, All authors approved the final version of the manuscript. **Sofia Amjad:** Writing – original draft, contributed to the artwork, drafted the paper, All authors approved the final version of the manuscript. **Mukhtiar Baig:** Writing – original draft, drafted the paper, All authors approved the final version of the manuscript. **Taseer Ahmed Khan:** critically reviewed the review, All authors approved the final version of the manuscript. **Rehana Rehman:** Conceptualization, Writing – original draft, conceptualized, designed, and drafted the review, contributed to the artwork, drafted the paper, All authors approved the final version of the manuscript.

## Declaration of competing interest

Authors have no conflict of interest to declare.

## References

[bib1] Agarwal A., Mulgund A., Hamada A., Chyatte M.R. (2015). A unique view on male infertility around the globe. Reprod. Biol. Endocrinol..

[bib2] Asadi N., Bahmani M., Kheradmand A., Rafieian-Kopaei M. (2017). The impact of oxidative stress on testicular function and the role of antioxidants in improving it: a review. J. Clin. Diagn. Res.: J. Clin. Diagn. Res..

[bib3] Bell E.L., Nagamori I., Williams E.O., Del Rosario A.M., Bryson B.D., Watson N. (2014). SirT1 is required in the male germ cell for differentiation and fecundity in mice. Development.

[bib4] Benayoun B.A., Georges A.B., L’Hote D., Andersson N., Dipietromaria A., Todeschini A.L. (2011). Transcription factor FOXL2 protects granulosa cells from stress and delays cell cycle: role of its regulation by the SIRT1 deacetylase. Hum. Mol. Genet..

[bib5] Bordone L., Motta M.C., Picard F., Robinson A., Jhala U.S., Apfeld J. (2006). Sirt1 regulates insulin secretion by repressing UCP2 in pancreatic beta cells. PLoS Biol..

[bib6] Brunet A., Sweeney L.B., Sturgill J.F., Chua K.F., Greer P.L., Lin Y. (2004). Stress-dependent regulation of FOXO transcription factors by the SIRT1 deacetylase. Science (New York, NY).

[bib8] Chandel N., Ayasolla K., Wen H., Lan X., Haque S., Saleem M.A. (2017). Vitamin D receptor deficit induces activation of renin angiotensin system via SIRT1 modulation in podocytes. Exp. Mol. Pathol..

[bib9] Coussens M., Maresh J.G., Yanagimachi R., Maeda G., Allsopp R. (2008). Sirt1 deficiency attenuates spermatogenesis and germ cell function. PloS One.

[bib10] Di Emidio G., Falone S., Vitti M., D’Alessandro A.M., Vento M., Di Pietro C. (2014). SIRT1 signalling protects mouse oocytes against oxidative stress and is deregulated during aging. Hum. Reprod. (Oxf.).

[bib11] Di Sante G., Wang L., Wang C., Jiao X., Casimiro M.C., Chen K. (2015). Sirt1-deficient mice have hypogonadotropic hypogonadism due to defective GnRH neuronal migration. Mol. Endocrinol..

[bib12] Donato A.J., Morgan R.G., Walker A.E., Lesniewski L.A. (2015). Cellular and molecular biology of aging endothelial cells. J. Mol. Cell. Cardiol..

[bib13] Gerber P.A., Rutter G.A. (2017). The role of oxidative stress and hypoxia in pancreatic beta-cell dysfunction in diabetes mellitus. Antioxidants Redox Signal..

[bib14] Go A.S., Mozaffarian D., Roger V.L., Benjamin E.J., Berry J.D., Borden W.B. (2013). Heart disease and stroke statistics--2013 update: a report from the American Heart Association. Circulation.

[bib15] Guan Y., Hao C.M. (2016). SIRT1 and kidney function. Kidney Dis..

[bib16] Hasegawa K., Wakino S., Yoshioka K., Tatematsu S., Hara Y., Minakuchi H. (2010). Kidney-specific overexpression of Sirt1 protects against acute kidney injury by retaining peroxisome function. J. Biol. Chem..

[bib17] He W., Wang Y., Zhang M.Z., You L., Davis L.S., Fan H. (2010). Sirt1 activation protects the mouse renal medulla from oxidative injury. J. Clin. Invest..

[bib18] Huang R., Xu Y., Wan W., Shou X., Qian J., You Z. (2015). Deacetylation of nuclear LC3 drives autophagy initiation under starvation. Mol. Cell.

[bib19] Hui X., Zhang M., Gu P., Li K., Gao Y., Wu D. (2017). Adipocyte SIRT1 controls systemic insulin sensitivity by modulating macrophages in adipose tissue. EMBO Rep..

[bib20] Hukshorn C.J., Lindeman J.H.N., Toet K.H., Saris W.H.M., Eilers P.H.C., Westerterp-Plantenga M.S. (2004). Leptin and the proinflammatory state associated with human obesity. J. Clin. Endocrinol. Metab..

[bib21] Kawamura Y., Uchijima Y., Horike N., Tonami K., Nishiyama K., Amano T. (2010). Sirt3 protects in vitro-fertilized mouse preimplantation embryos against oxidative stress-induced p53-mediated developmental arrest. J. Clin. Invest..

[bib22] Kilic U., Gok O., Elibol-Can B., Ozgen I.T., Erenberk U., Uysal O. (2015). SIRT1 gene variants are related to risk of childhood obesity. Eur. J. Pediatr..

[bib23] Kitada M., Ogura Y., Koya D. (2016). The protective role of Sirt1 in vascular tissue: its relationship to vascular aging and atherosclerosis. Aging.

[bib24] Kolthur-Seetharam U., Teerds K., de Rooij D.G., Wendling O., McBurney M., Sassone-Corsi P. (2009). The histone deacetylase SIRT1 controls male fertility in mice through regulation of hypothalamic-pituitary gonadotropin signaling. Biol. Reprod..

[bib25] Kolthur-Seetharam U., Teerds K., de Rooij D.G., Wendling O., McBurney M., Sassone-Corsi P. (2009). The histone deacetylase SIRT1 controls male fertility in mice through regulation of hypothalamic-pituitary gonadotropin signaling. Biol. Reprod..

[bib26] Leber B., Lin J., Andrews D.W. (2007). Embedded together: the life and death consequences of interaction of the Bcl-2 family with membranes. Apoptosis.

[bib27] Li D., Dammer E.B., Sewer M.B. (2012). Resveratrol stimulates cortisol biosynthesis by activating SIRT-dependent deacetylation of P450scc. Endocrinology.

[bib28] Li L., Wei W., Zhang Y., Tu G., Zhang Y., Yang J. (2015). SirT1 and STAT3 protect retinal pigmented epithelium cells against oxidative stress. Mol. Med. Rep..

[bib29] Li S., Hong H., Lv H., Wu G., Wang Z. (2016). SIRT 1 overexpression is associated with metastasis of pancreatic ductal adenocarcinoma (PDAC) and promotes migration and growth of PDAC cells. Med. Sci. Mon. Int. Med. J. Exp. Clin. Res.: international medical journal of experimental and clinical research.

[bib30] Liang Q.X., Lin Y.H., Zhang C.H., Sun H.M., Zhou L., Schatten H. (2018). Resveratrol increases resistance of mouse oocytes to postovulatory aging in vivo. Aging.

[bib31] Liu J., Liu M., Ye X., Liu K., Huang J., Wang L. (2012). Delay in oocyte aging in mice by the antioxidant N-acetyl-L-cysteine (NAC). Hum. Reprod. (Oxf.).

[bib32] Liu Y., He X.Q., Huang X., Ding L., Xu L., Shen Y.T. (2013). Resveratrol protects mouse oocytes from methylglyoxal-induced oxidative damage. PloS One.

[bib33] Liu C., Song Z., Wang L., Yu H., Liu W., Shang Y. (2017). Sirt1 regulates acrosome biogenesis by modulating autophagic flux during spermiogenesis in mice. Development.

[bib34] Luo X.Y., Qu S.L., Tang Z.H., Zhang Y., Liu M.H., Peng J. (2014). SIRT1 in cardiovascular aging. Clin. Chim. Acta.

[bib35] Luo G., Jian Z., Zhu Y., Zhu Y., Chen B., Ma R. (2019). Sirt1 promotes autophagy and inhibits apoptosis to protect cardiomyocytes from hypoxic stress. Int. J. Mol. Med..

[bib36] McBurney M.W., Yang X., Jardine K., Hixon M., Boekelheide K., Webb J.R. (2003). The mammalian SIR2alpha protein has a role in embryogenesis and gametogenesis. Mol. Cell Biol..

[bib37] Merksamer P.I., Liu Y., He W., Hirschey M.D., Chen D., Verdin E. (2013). The sirtuins, oxidative stress and aging: an emerging link. Aging.

[bib38] Miranda-Vizuete A., Damdimopoulos A.E., Spyrou G.J.A., signaling r (2000). The mitochondrial thioredoxin system.

[bib39] Miyazaki R., Ichiki T., Hashimoto T., Inanaga K., Imayama I., Sadoshima J. (2008). SIRT1, a longevity gene, downregulates angiotensin II type 1 receptor expression in vascular smooth muscle cells. Arterioscler. Thromb. Vasc. Biol..

[bib40] Morgan D., Oliveira-Emilio H.R., Keane D., Hirata A.E., Santos da Rocha M., Bordin S. (2007). Glucose, palmitate and pro-inflammatory cytokines modulate production and activity of a phagocyte-like NADPH oxidase in rat pancreatic islets and a clonal beta cell line. Diabetologia.

[bib42] Morita Y., Wada-Hiraike O., Yano T., Shirane A., Hirano M., Hiraike H. (2012). Resveratrol promotes expression of SIRT1 and StAR in rat ovarian granulosa cells: an implicative role of SIRT1 in the ovary.

[bib43] Morris B.J. (2013). Seven sirtuins for seven deadly diseases of aging. Free Radical Biol. Med..

[bib44] Mostafa T., Rashed L., Osman I., Marawan M. (2015). Seminal plasma oxytocin and oxidative stress levels in infertile men with varicocele. Andrologia.

[bib45] Mostafa T., Nabil N., Rashed L., Makeen K., El-Kasas M., Mohamaed H. (2018). Seminal SIRT 1 expression in infertile oligoasthenoteratozoospermic men with varicocoele. Andrology.

[bib46] Moynihan K.A., Grimm A.A., Plueger M.M., Bernal-Mizrachi E., Ford E., Cras-Méneur C. (2005). Increased dosage of mammalian Sir2 in pancreatic β cells enhances glucose-stimulated insulin secretion in mice. Cell Metabol..

[bib47] Mutch D.M., Clément K. (2006). Genetics of human obesity. Best Pract. Res. Clin. Endocrinol. Metabol..

[bib48] Nakao M., Anan K., Araki H., Hino SJTiE. (2019). Metabolism. Distinct roles of the NAD+-Sirt1 and FAD-LSD1 pathways in metabolic response and tissue development.

[bib49] Nakata R., Takahashi S., Inoue H. (2012). Recent advances in the study on resveratrol. Biol. Pharm. Bull..

[bib50] Nemoto S., Fergusson M.M., Finkel T. (2005). SIRT1 functionally interacts with the metabolic regulator and transcriptional coactivator PGC-1{alpha}. J. Biol. Chem..

[bib53] Ota H., Eto M., Ogawa S., Iijima K., Akishita M., Ouchi Y. (2010). SIRT1/eNOS axis as a potential target against vascular senescence, dysfunction and atherosclerosis. J. Atherosclerosis Thromb..

[bib54] Ou X., Lee M.R., Huang X., Messina-Graham S., Broxmeyer H.E. (2014). SIRT1 positively regulates autophagy and mitochondria function in embryonic stem cells under oxidative stress. Stem Cell..

[bib55] Peck B., Chen C.-Y., Ho K.-K., Di Fruscia P., Myatt S.S., Coombes R.C. (2010). SIRT inhibitors induce cell death and p53 acetylation through targeting both SIRT1 and SIRT2. Mol. Canc. Therapeut..

[bib56] Picard F., Kurtev M., Chung N., Topark-Ngarm A., Senawong T., Machado De Oliveira R. (2004). Sirt1 promotes fat mobilization in white adipocytes by repressing PPAR-gamma. Nature.

[bib57] Pillarisetti S. (2008). A review of Sirt1 and Sirt1 modulators in cardiovascular and metabolic diseases. Recent Pat. Cardiovasc. Drug Discov..

[bib58] Ramadori G., Fujikawa T., Fukuda M., Anderson J., Morgan D.A., Mostoslavsky R. (2010). SIRT1 deacetylase in POMC neurons is required for homeostatic defenses against diet-induced obesity. Cell Metabol..

[bib59] Rehman R., Abidi S.H., Alam F. (2018). Metformin, oxidative stress and infertility: a way forward. Front. Physiol..

[bib60] Rodgers J.T., Lerin C., Haas W., Gygi S.P., Spiegelman B.M., Puigserver P. (2005). Nutrient control of glucose homeostasis through a complex of PGC-1alpha and SIRT1. Nature.

[bib62] Sirotkin A.V., Dekanova P., Harrath A.H., Alwasel S.H., Vasicek D. (2014). Interrelationships between sirtuin 1 and transcription factors p53 and NF-kappaB (p50/p65) in the control of ovarian cell apoptosis and proliferation. Cell Tissue Res..

[bib64] St-Pierre J., Drori S., Uldry M., Silvaggi J.M., Rhee J., Jager S. (2006). Suppression of reactive oxygen species and neurodegeneration by the PGC-1 transcriptional coactivators. Cell.

[bib65] Szendroedi J., Phielix E., Roden M. (2011). The role of mitochondria in insulin resistance and type 2 diabetes mellitus. Nat. Rev. Endocrinol..

[bib66] Tao X., Zhang X., Ge S.Q., Zhang E.H., Zhang B. (2015). Expression of SIRT1 in the ovaries of rats with polycystic ovary syndrome before and after therapeutic intervention with exenatide. Int. J. Clin. Exp. Pathol..

[bib67] Tatone C., Di Emidio G. (2015).

[bib68] Tatone C., Di Emidio G., Barbonetti A., Carta G., Luciano A.M., Falone S. (2018). Sirtuins in gamete biology and reproductive physiology: emerging roles and therapeutic potential in female and male infertility. Hum. Reprod. Update.

[bib69] Tatone C., Di Emidio G., Barbonetti A., Carta G., Luciano A.M., Falone S. (2018). Sirtuins in gamete biology and reproductive physiology: emerging roles and therapeutic potential in female and male infertility. Hum. Reprod. Update.

[bib70] Toorie A.M., Cyr N.E., Steger J.S., Beckman R., Farah G., Nillni E.A. (2016). The nutrient and energy sensor Sirt1 regulates the hypothalamic-pituitary-adrenal (HPA) Axis by altering the production of the prohormone convertase 2 (PC2) essential in the maturation of corticotropin-releasing hormone (CRH) from its prohormone in male rats. J. Biol. Chem..

[bib71] Wang Y.Q., Cao Q., Wang F., Huang L.Y., Sang T.T., Liu F. (2015). SIRT1 protects against oxidative stress-induced endothelial progenitor cells apoptosis by inhibiting FOXO3a via FOXO3a ubiquitination and degradation. J. Cell. Physiol..

[bib72] Xiong S., Salazar G., Patrushev N., Alexander RWJJoBC (2011). FoxO1 mediates an autofeedback loop regulating SIRT1 expression.

[bib73] Xu H., Barnes G.T., Yang Q., Tan G., Yang D., Chou C.J. (2003). Chronic inflammation in fat plays a crucial role in the development of obesity-related insulin resistance. J. Clin. Invest..

[bib74] Yamamoto M., Takahashi Y. (2018). The essential role of SIRT1 in hypothalamic-pituitary Axis. Front. Endocrinol..

[bib75] Ye J., Liu Z., Wei J., Lu L., Huang Y., Luo L. (2013). Protective effect of SIRT1 on toxicity of microglial-derived factors induced by LPS to PC12 cells via the p53-caspase-3-dependent apoptotic pathway.

[bib76] Zhang D., Li S., Cruz P., Kone B.C. (2009). Sirtuin 1 functionally and physically interacts with disruptor of telomeric silencing-1 to regulate alpha-ENaC transcription in collecting duct. J. Biol. Chem..

[bib77] Zhang H.H., Ma X.J., Wu L.N., Zhao Y.Y., Zhang P.Y., Zhang Y.H. (2015). SIRT1 attenuates high glucose-induced insulin resistance via reducing mitochondrial dysfunction in skeletal muscle cells. Exp. Biol. Med..

[bib78] Zhang W., Huang Q., Zeng Z., Wu J., Zhang Y., Chen Z. (2017). Sirt1 inhibits oxidative stress in vascular endothelial cells. Oxidative Medicine and Cellular Longevity.

[bib79] Zhou L., Wang S.I., Moon Y.J., Kim K.M., Lee K.B., Park B.H. (2017). Overexpression of SIRT1 prevents hypoxia-induced apoptosis in osteoblast cells. Mol. Med. Rep..

